# Negative symptoms and social cognition as mediators of the relationship between neurocognition and functional outcome in schizophrenia

**DOI:** 10.1192/j.eurpsy.2025.210

**Published:** 2025-08-26

**Authors:** A. Mucci

**Affiliations:** Mental and Physical Health and Preventive Medicine, University of Campania L. Vanvitelli, Naples, Italy

## Abstract

**Abstract:**

The presentation will summarize recent findings on the role of the two domains of negative symptoms (motivational impairment and expressive impairment) and social cognition as mediators of the impact of neurocognitive impairment on functional outcomes in people living with schizophrenia (PLWS).

One hundred and fifty subjects from 8 different European centers were recruited. Our data showed that the expressive impairment predicted global functioning and together with motivational impairment fully mediated the effects of neurocognition on the same outcome. Motivational impairment was a predictor of personal and social functioning and fully mediated neurocognitive impairment effects on the same outcome. Both motivational and neurocognitive impairments predicted socially useful activities, and the emotion recognition domain of social cognition partially mediated the impact of neurocognitive deficits on this outcome.

Our results indicate that pathways to functional outcomes are specific for different domains of real-life functioning and that negative symptoms and social cognition mediate the impact of neurocognitive impairment on different domains of functioning. Our results suggest that psychosocial interventions should target both negative symptoms and social cognition to enhance the functional impact of cognitive remediation.

**Disclosure of Interest:**

A. Mucci Consultant of: Angelini, Gedeon Richter Bulgaria, Janssen Pharmaceuticals, Lundbeck, Otsuka Pharmaceutical, Pfizer, Pierre Fabre, Rovi Pharma and Boehringer Ingelheim.

